# Videoconference-Supervised Group Exercise Reduces Low Back Pain in Eldercare Workers: Results from the ReViEEW Randomised Controlled Trial

**DOI:** 10.1007/s10926-024-10182-2

**Published:** 2024-04-17

**Authors:** Ander Espin, Jon Irazusta, Maialen Aiestaran, Unai Latorre Erezuma, Julia García-García, Ismene Arrinda, Karmele Acedo, Ana Rodriguez-Larrad

**Affiliations:** 1 Ageing On Research Group, Department of Physiology, University of the Basque Country (UPV/EHU), Leioa, Spain; 2 Biobizkaia Health Research Institute, Barakaldo, Spain; 3 IMQ Igurco Residencias Sociosanitarias, Bilbao, Spain; 4 Home Care Lab, S. Coop, Bilbao, Spain

**Keywords:** Musculoskeletal pain, Telerehabilitation, Resistance training, Occupational health, Quality of life

## Abstract

**Purpose:**

To assess the effects of a group exercise intervention conducted by real-time videoconference on the low back pain of eldercare workers.

**Methods:**

We randomly assigned 130 eldercare workers to an experimental group (EG: *n* = 65) or control group (CG: *n* = 65). Participants from both groups took part in routine prevention programs carried out in their workplace, and participants from the EG received an additional 12-week resistance-exercise intervention supervised by real-time videoconference. Assessments were conducted before and after the intervention, and the primary outcome was average low back pain intensity during the last 7 days, measured by the 0–10 numerical rating scale. Secondary outcomes included additional measures of low back, neck, shoulder and hand/wrist pain, as well as psycho-affective parameters, medication consumption and muscle performance. Both intention-to-treat and per-protocol analyses were applied with a group-by-time ANCOVA including baseline measurements as covariates.

**Results:**

125 participants completed post-intervention assessments (EG: *n* = 63, CG: *n* = 62). The intention-to-treat analysis showed an effect favouring the EG on average low back pain intensity (*p* = 0.034). Improvements in additional low back and hand/wrist pain outcomes were also observed, as well as on upper limb muscle performance (*p* < 0.05). The per-protocol analysis demonstrated additional benefits in depression, quality of life, hypnotic/anxiolytic medication consumption and lower limb and trunk muscle performance in participants with ≥ 50% adherence (*p* < 0.05).

**Conclusions:**

The intervention was effective for reducing the low back and hand/wrist pain of eldercare workers and increasing upper limb muscle performance. The per-protocol analysis showed additional benefits in psycho-affective parameters, medication consumption and muscle performance.

**Trial registration:** ClinicalTrials.gov, NCT05050526. Registered 20 September 2021—Prospectively registered, https://www.clinicaltrials.gov/study/NCT05050526

**Supplementary Information:**

The online version contains supplementary material available at 10.1007/s10926-024-10182-2.

## Introduction

Eldercare workers are qualified professionals who provide assistance with activities of daily living to dependent older people at either home or long-term facilities. These workers are of paramount importance in current and future society, as eldercare needs are increasing sharply due to the ageing of the population [1]. Eldercare work is characterized by high physically [2] and psychologically [3] demanding tasks, which can contribute to the development of musculoskeletal pain [4]. Although the presence of pain in the neck, shoulders and the upper extremity is also common, low back pain is the most frequent musculoskeletal disorder among eldercare workers [5]. Low back pain is the leading cause of disability and productivity loss worldwide [6], and it can severely affect the quality of life of the people who suffer from it [7]. Pain in the low back is often associated with mental health problems [8] and sleep [9] disturbances, which can lead to higher levels of disability, worse recovery, and greater primary healthcare utilization [10].

Current literature supports exercise as a tool with solid evidence in pain management [11]. A recent review concluded that therapeutic exercise is strongly recommended in chronic low back pain, as it has the potential to decrease pain, improve function and reduce disability [12]. There is also evidence suggesting that exercise could mitigate the psychological disorders associated to musculoskeletal pain [13]. Tele-rehabilitation is an increasingly used modality for delivering exercise interventions remotely [14]. In comparison with in-person programs, tele-exercise could be logistically and economically advantageous, as time and money costs associated with displacements are avoided [15]. Moreover, tele-rehabilitation is compatible with situations in which interpersonal physical distancing measures are required, such as the recent COVID-19 pandemic [16].

To date, the majority of tele-rehabilitation interventions have consisted of websites for autonomous consultation, thus lacking real-time supervision [14]. Among the few interventions with real-time supervision, most have followed an individual approach (e.g., individual videoconference sessions with the therapist supervising a single participant at a time) [17]. Real-time supervision and group dynamic can lead to higher participant adherence [18, 19], what could result in greater effectiveness of the intervention. Moreover, real-time supervision could be beneficial in terms of safety, as participants are continuously monitored. However, group tele-exercise interventions with real-time supervision have been scarce [17] and, to our knowledge, none of them has been carried out in the workplace.

Therefore, we conceived the ReViEEW trial (acronym for “Real-time Videoconference-based Exercise in Eldercare Workers”), which assessed, to our knowledge, the first group tele-exercise intervention with synchronous supervision in the occupational setting. The primary aim of the study was to assess the effects of the intervention on the low back pain of eldercare workers. Secondary outcomes included additional measures of musculoskeletal pain, psycho-affective parameters, hypnotic/anxiolytic and pain medication consumption and muscle performance.

## Methods

### Study Design

A parallel-assignment, two-arm, multicentre randomised controlled trial (RCT) was carried out. The study was designed so that both the assessments and the intervention could be conducted remotely via real-time videoconference. The overall study protocol is described elsewhere [20]. For participant recruitment, institutions offering eldercare services at home or in long-term facilities and located in the Basque Country (Spain) were contacted following non-probabilistic procedures. At each of the institutions that were interested in participating, all eldercare workers who met the selection criteria were invited to complete the baseline assessments. Following baseline measurements, participants were randomly assigned (1:1 ratio) in each institution through sealed opaque envelopes to either an experimental or control group by a coin-tossing sequence generation. Assessments were conducted at baseline and at the end of the intervention. Outcome assessors and researchers performing data analysis were blinded to group allocation. The study protocol was approved by the Ethics Committee for Research Involving Human Beings of the University of the Basque Country (M10/2019/200) and prospectively registered at ClinicalTrials.gov (NCT05050526). Informed written consent was obtained from all participants before enrolling in the study.

### Participants

Participants had to meet all the following criteria to be eligible for the study: (a) formal eldercare worker on active duty, (b) ≥ 18 years of age, (c) ≥ 3 months of experience in the profession, and (d) employment contract until at least the date of study completion. Participants were excluded if (a) they were pregnant or (b) their participation was considered contraindicated according to the exercise preparticipation health screening guidelines by the American College of Sports Medicine [21].

### Control Group

Participants from the control group took part in the routine prevention programs carried out in their corresponding institutions, which mainly consisted of regular group-based training on manual and technical aid-assisted patient handling. These training activities were all held in the workplace, led by a physiotherapist, carried out annually with a duration of around 20 h, and combined theoretical classes (e.g., concepts about how to do manual and technical aid-assisted transfers to dependent elderly people in biomechanically correct postures) and practical exercises (e.g., role-playing among eldercare workers to put the concepts learned into practice).

### Experimental Group

In addition to the aforementioned prevention programs, participants from the experimental group took part in a 12-week exercise intervention, consisting of two sessions per week of 45 min each. Sessions were carried out in small groups of ≤ 10 participants, in the workplace but outside working hours, and remotely supervised by the instructor using real-time videoconference (Supplementary Information, Fig. SI1). Sessions started with a warm-up (5–10 min) consisting of joint mobility and aerobic activation exercises to increase heart rate. The main part of the session consisted of resistance exercises performed with body-weight and elastic bands (30 min). A total of 9 exercises were performed throughout the program (Supplementary Information, Fig. SI2). In each session, 4 sets of 6 exercises were performed. Exercises were systematically varied between sessions so that each of them was evenly performed during the whole program. In each set, exercises for the major muscle groups were alternated in a circuit format (e.g., upper limb, lower limb, trunk, upper limb, lower limb, trunk). For each exercise, three levels of progression were established: progression 1 (weeks 1–4), progression 2 (weeks 5–8) and progression 3 (weeks 9–12). Progression was achieved by modifying the exercise technique or using elastic bands of different resistance. Within each level of progression, the work:rest time ratio devoted to each exercise also increased from 30:30 to 45:15 s (Supplementary Information, Fig. SI3). Participants were monitored to reach an intensity between 3 (moderate) and 5 (strong) on the Borg’s CR-10 scale [22] and not to reach failure on any of the exercises. If an exercise caused intolerable pain, the 4-stage exercise adjustment model suggested by Jakobsen et al. was used [23]. Sessions finished with a cool-down (5–10 min) consisting of static stretching and breathing/relaxing exercises.

### Adherence and Adverse Events

In each session, the instructor collected the following information from each participant: attendance, session completion, and overall perceived intensity during the session. Adherence to the intervention was defined as the percentage of sessions in which participants performed the planned training regarding completion and intensity (i.e., 24 sessions completed with perceived intensity between 3 and 5 in Borg’s CR-10 scale = 100% adherence). The instructor also collected adverse events occurring during the sessions. Adverse events were divided into two types: technical (connection and/or operation problems with the videoconference system) or participant safety-related (pain, discomfort, or any other health-related problem). They were also classified as minor (those slightly hindering the development of the session) or major (those preventing the development of the session).

### Baseline Descriptive Data

Participants reported the following descriptive data at baseline: date of birth, sex, height and weight, marital status, educational level, number of children and presence of children cohabiting at home, care for dependent people outside the work environment, weekly working hours, years of experience in the profession, presence of rotative and night work shifts, alcohol and tobacco consumption, compliance with World Health Organization’s physical activity guidelines, and practice of regular resistance training.

### Primary Outcome

The primary outcome was average pain intensity in the low back during the last 7 days, measured by an 11-point numerical rating scale (NRS) ranging from 0 (complete absence of pain) to 10 (worst imaginable pain) [24]. The NRS is a valid, reliable, and widely used tool for the measurement of pain intensity, which has been proposed as the most appropriate for research purposes in comparison with other pain scales [24].

### Secondary Outcomes

#### Musculoskeletal Pain

Musculoskeletal pain outcomes referring to the last 7 days were collected separately for the low back, neck, shoulders, and hands/wrists. Average and worst intensity (0–10) were measured by the aforementioned 11-point NRS [24]. Frequency was defined as the number of days in pain (0–7), and interference as the number of days in which pain negatively interfered with work (0–7).

Additionally, participants reported the number of days in which they took pain medication during the last 7 days (0–7).

#### Psycho-Affective Parameters

Happiness was measured by the subjective happiness scale [25]. It consists of four items in a 7-point Likert response format asking about current perceived happiness. A single composite score is obtained by averaging responses to the four items, and higher values indicate greater happiness.

Anxiety and depression were measured by Goldberg’s scales [26]. They consist of two separate scales containing nine dichotomized (yes/no) response items each, asking about last month’s anxious and depressive symptoms, respectively. Higher scores indicate greater anxiety/depression levels.

Quality of life was measured by the EuroQol-5D 0–100 health state scale [27]. It consists of a single item measuring self-perceived current health state in a scale ranging from 0 (worst imaginable) to 100 (best imaginable).

Sleep quality was assessed by the single-item sleep quality scale [28]. It measures overall sleep quality during the last 7 days in a numerical scale ranging from 0 (terrible) to 10 (excellent).

Additionally, participants reported the number of days of hypnotic/anxiolytic medication consumption during the last 7 days.

#### Muscle Performance

The following muscle performance tests were carried out in the same modality as the exercise sessions (i.e., with the participant located at the workplace and the assessor remotely supervising the execution of the tests via real-time videoconference). The tests were previously validated to be carried out remotely, showing they are feasible and reliable when conducted by videoconference [29].

The 5-repetition sit to stand test was used to assess lower limbs’ muscle performance. Participants had to stand up from and sit down on a chair five times as quickly as possible. The time taken to complete the five repetitions is registered and reported in seconds, with shorter times indicating better performance. The mean of two attempts was registered.

The kneeling push-up test was used to assess upper limbs’ muscle performance. Participants had to do the maximum number of push-ups possible using the knees as the pivotal point. The total number of repetitions is registered, with more repetitions indicating better performance.

The Shirado-Ito trunk flexor endurance test was used to assess trunk’s muscle performance. Participants had to maintain a defined trunk flexion position for as long as possible. The total time was registered in seconds, with longer times indicating better performance.

### Sample Size Calculation

The sample size was calculated to detect a change in low back pain that could be relevant in terms of work absenteeism [30]. Considering the average low back pain intensity of 5.0 (SD 2.6) in the 11-point NRS observed in a previous study carried out by our research group in eldercare workers [31], and accepting an alpha error of 0.05 and a beta error of 0.20 in a bilateral contrast, 108 participants were needed to detect a difference of ≥ 1 unit. Due to expected dropouts, the sample size was increased by 20%. Consequently, the required sample was 130 participants (*n* = 65 in the experimental and *n* = 65 in the control groups, respectively).

### Statistical Analysis

Data analysis was performed with IBM SPSS Statistics 27 statistical software package (SPSS, Inc., Chicago, IL). Continuous data are expressed as means with standard deviations (SD), and categorical variables as frequency counts and percentages (%). Normality of distribution was assessed with the Shapiro–Wilk and Kolmogorov–Smirnov tests for samples < 50 and ≥ 50, respectively. Non-normally distributed variables were square-root transformed for statistical analyses. Between-group baseline differences were analysed with the independent samples T and Chi-squared tests for continuous and categorical variables, respectively. Effects of the intervention were assessed with a group-by-time ANCOVA including baseline measurements as covariates, and effect size was estimated by partial eta squared (η_p_^2^). Values for η_p_^2^ of 0.01, 0.06, and 0.14 were considered small, medium and large, respectively [32]. As initially planned, the primary analysis was based on intention-to-treat (ITT). A per-protocol (PP) analysis was also performed including only participants with ≥ 50% adherence to the intervention. Additionally, a post-hoc subgroup analysis was performed to assess the effects of the intervention on low back pain outcomes separately in participants with (≥ 1 in average 11-point NRS) and without (< 1 in average 11-point NRS) low back pain at baseline. The level of statistical significance was set at *p* < 0.05.

## Results

### Participants

**Fig. 1:**
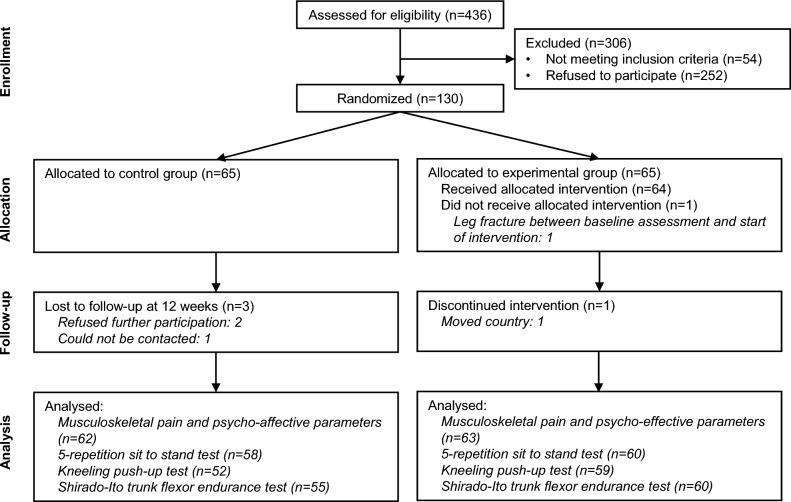
CONSORT flow diagram

A total of 130 participants were recruited and randomised to the experimental (*n* = 65) and control (*n* = 65) groups (Fig. 1). Participants were recruited from five long-term nursing homes (*n* = 11, *n* = 12, *n* = 14, *n* = 16, and *n* = 27, respectively) and one institution providing at-home eldercare services (*n* = 50). There were not significant differences at baseline between the experimental and control groups (*p* > 0.05) (Table 1). Two participants from the experimental group and three participants from the control group were lost to follow-up. Study start dates differed in each of the institutions. Overall, the first participant was recruited in October 2021, and the last 12-week follow-up was in June 2023.

**Table 1:** Baseline characteristics of the participants

Characteristic	Exp (*n* = 65)	Con (*n* = 65)
Age (years), mean (SD)	49 (9)	50 (9)
Sex, *n* females (%)	61 (94)	63 (97)
Height (cm), mean (SD)	163 (7)	162 (7)
Weight (kg), mean (SD)	67 (11)	69 (15)
Body mass index (kg/m^2^), mean (SD)	25.4 (4.4)	26.5 (5.3)
Married, *n* yes (%)	35 (54)	38 (59)
Children, *n* yes (%)	45 (69)	48 (74)
Children cohabiting at home, *n* yes (%)	38 (58)	39 (60)
Care for dependent people outside work, *n* yes (%)	14 (22)	12 (19)
Secondary or higher education, *n* yes (%)	61 (94)	57 (88)
Experience in profession (years), mean (SD)	16 (10)	15 (9)
Working hours (hours/week), mean (SD)	33 (5)	32 (7)
Rotative work shift, *n* yes (%)	29 (45)	35 (54)
Night work shift, *n* yes (%)	12 (19)	13 (20)
Alcohol consumption, *n* yes (%)	45 (69)	50 (77)
Tobacco consumption, *n* yes (%)	24 (37)	19 (29)
Meet WHO physical activity guidelines, *n* yes (%)	26 (40)	21 (32)
Regular resistance training, *n* yes (%)	14 (22)	9 (14)

*Con* control group, *Exp* experimental group, *WHO* World Health Organization, no differences between groups (*p* > 0.05)

### Adherence and Adverse Events

One single physical therapist with previous experience conducting group therapeutic exercise programs delivered all the sessions. Mean adherence to the intervention was 67% (SD 31%). Mean number of participants in each session was 4.3 (SD 2.3). There were minor technical and participant safety-related adverse events in 31 (12%) and 24 (9%) sessions, respectively. Technical adverse events were mainly connection problems which slightly hindered communication. The only major adverse event was a connection drop in a centre that prevented the development of the session one day. Participant safety-related adverse events were musculoskeletal pains that required exercise adjustment.

### Intervention Effects: ITT Analysis

The group-by-time ANCOVA showed a significant effect of the intervention favouring the experimental group on average low back pain intensity (*p* = 0.034) (Table 2).

**Table 2:** Effects of the intervention: intention-to-treat (ITT) analysis

Outcome	Experimental		Control		ANCOVA (*p*)	η_p_^2^
	Pre	Post	Pre	Post		
Low back pain						
Average intensity (0–10)	3.4 (2.4)	2.4 (2.2)	3.0 (2.4)	3.1 (2.5)	**0.034**	0.037
Worst intensity (0–10)	4.1 (2.9)	3.1 (2.8)	3.7 (3.0)	3.8 (3.0)	0.080	0.025
Frequency (0–7)	3.0 (2.4)	1.9 (1.9)	2.7 (2.2)	2.8 (2.4)	**0.010**	0.054
Interference (0–7)	1.3 (2.1)	0.9 (1.6)	1.2 (1.8)	1.7 (2.0)	**0.001**	0.082
Neck pain						
Average intensity (0–10)	3.0 (2.9)	2.3 (2.6)	3.4 (3.1)	2.9 (2.6)	0.163	0.016
Worst intensity (0–10)	3.4 (3.2)	2.7 (2.8)	4.0 (3.4)	3.4 (3.0)	0.187	0.014
Frequency (0–7)	2.7 (2.5)	1.8 (2.2)	2.9 (2.6)	2.6 (2.4)	0.058	0.029
Interference (0–7)	1.5 (1.2)	1.2 (2.0)	1.5 (2.1)	1.7 (2.3)	0.137	0.018
Shoulder pain						
Average intensity (0–10)	2.5 (2.6)	2.0 (2.5)	3.0 (2.9)	2.4 (2.7)	0.830	< 0.001
Worst intensity (0–10)	2.7 (2.8)	2.3 (2.9)	3.7 (3.3)	2.8 (3.0)	0.943	< 0.001
Frequency (0–7)	2.3 (2.4)	1.8 (2.3)	2.9 (2.6)	2.2 (2.4)	0.788	0.001
Interference (0–7)	1.3 (2.0)	1.1 (2.0)	1.6 (2.1)	1.6 (2.2)	0.370	0.007
Hand/wrist pain						
Average intensity (0–10)	2.2 (2.6)	1.3 (2.1)	3.0 (3.1)	2.5 (2.9)	**0.023**	0.041
Worst intensity (0–10)	2.4 (2.7)	1.5 (2.3)	3.3 (3.5)	3.1 (3.4)	**0.017**	0.046
Frequency (0–7)	1.8 (2.2)	1.3 (1.9)	2.6 (2.8)	2.5 (2.7)	**0.035**	0.036
Interference (0–7)	1.2 (1.9)	0.9 (1.7)	1.9 (2.5)	1.9 (2.4)	**0.049**	0.031
Pain medication (0–7)	2.4 (2.4)	2.3 (2.5)	2.2 (2.6)	2.6 (2.7)	0.124	0.019
Psycho-affective parameters						
Happiness (1–7)	5.4 (1.0)	5.5 (1.0)	5.4 (0.8)	5.3 (0.9)	0.211	0.013
Anxiety (0–9)	4.0 (2.5)	3.4 (2.6)	4.2 (2.7)	3.7 (2.7)	0.705	0.001
Depression (0–9)	2.1 (2.0)	1.8 (1.8)	2.1 (2.1)	2.0 (1.7)	0.123	0.019
Quality of life (0–100)	75 (14)	80 (16)	70 (16)	72 (17)	0.053	0.030
Sleep quality (0–10)	6.1 (2.2)	6.5 (2.3)	6.1 (1.8)	6.5 (2.0)	0.886	< 0.001
Hypnotic/anxiolytic medication (0–7)	1.1 (2.3)	0.7 (2.1)	0.9 (2.2)	1.1 (2.4)	0.069	0.027
Muscle performance						
5-repetition sit to stand test (s)	7.4 (2.3)	7.0 (2.1)	7.5 (2.0)	7.5 (2.2)	0.072	0.028
Kneeling push-up test (repetitions)	3.5 (4.9)	5.6 (6.4)	4.3 (5.5)	4.6 (6.4)	**0.040**	0.039
Shirado-Ito trunk flexor test (s)	47 (33)	54 (28)	52 (45)	50 (37)	0.077	0.028

Bold values are statistically significant (*p* < 0.05)

Data are mean (SD)

*s* seconds, no between-group differences in Pre (*p* > 0.05)

There were also significant group-by-time interactions in favour of the experimental group on low back pain frequency (*p* = 0.010) and interference (*p* = 0.001), as well as on all hand/wrist pain outcomes: average intensity (*p* = 0.023), worst intensity (*p* = 0.017), frequency (*p* = 0.035) and interference (*p* = 0.049).

Respecting muscle performance, there was a significant group-by-time effect favouring the experimental group in the kneeling push-up test (*p* = 0.040).

A summary of the effect sizes of the outcomes showing statistically significant group-by-time interactions can be found in the Supplementary Information (Table SI1).

There were not significant group-by-time interactions on the remaining variables (*p* > 0.05).

### Intervention Effects: PP Analysis

Forty-eight participants (74%) from the experimental group had a ≥ 50% adherence to the intervention. When including only those participants in the analysis, the group-by-time ANCOVA also showed a significant effect of the intervention favouring the experimental group on average low back pain intensity (*p* = 0.011) (Table 3).

**Table 3:** Effects of the intervention: per-protocol (PP) analysis

Outcome	Experimental		Control		ANCOVA (*p*)	η_p_^2^
	Pre	Post	Pre	Post		
Low back pain						
Average intensity (0–10)	3.0 (2.5)	1.9 (2.0)	3.0 (2.4)	3.1 (2.5)	**0.011**	0.058
Worst intensity (0–10)	3.7 (2.9)	2.6 (2.5)	3.7 (3.0)	3.8 (3.0)	**0.036**	0.040
Frequency (0–7)	2.6 (2.4)	1.4 (1.7)	2.7 (2.2)	2.8 (2.4)	**0.003**	0.082
Interference (0–7)	0.9 (1.7)	0.6 (1.2)	1.2 (1.8)	1.7 (2.0)	** < 0.001**	0.108
Neck pain						
Average intensity (0–10)	2.8 (2.9)	2.1 (2.4)	3.4 (3.1)	2.9 (2.6)	0.106	0.024
Worst intensity (0–10)	3.3 (3.2)	2.5 (2.7)	4.0 (3.4)	3.4 (3.0)	0.117	0.023
Frequency (0–7)	2.5 (2.4)	1.6 (1.9)	2.9 (2.6)	2.6 (2.4)	**0.039**	0.039
Interference (0–7)	1.4 (2.2)	1.0 (1.7)	1.5 (2.1)	1.7 (2.3)	0.093	0.026
Shoulder pain						
Average intensity (0–10)	2.4 (2.7)	1.8 (2.4)	3.0 (2.9)	2.4 (2.7)	0.475	0.005
Worst intensity (0–10)	2.6 (2.9)	2.1 (2.9)	3.7 (3.3)	2.8 (3.0)	0.700	0.001
Frequency (0–7)	2.4 (2.5)	1.5 (2.1)	2.9 (2.6)	2.2 (2.4)	0.341	0.008
Interference (0–7)	1.3 (2.2)	0.9 (1.8)	1.6 (2.1)	1.6 (2.2)	0.113	0.023
Hand/wrist pain						
Average intensity (0–10)	2.4 (2.6)	1.3 (2.2)	3.0 (3.1)	2.5 (2.9)	**0.010**	0.060
Worst intensity (0–10)	2.6 (2.8)	1.5 (2.4)	3.3 (3.5)	3.1 (3.4)	**0.009**	0.061
Frequency (0–7)	2.0 (2.2)	1.3 (1.9)	2.6 (2.8)	2.5 (2.7)	**0.023**	0.047
Interference (0–7)	1.3 (2.0)	0.9 (1.8)	1.9 (2.5)	1.9 (2.4)	0.058	0.033
Pain medication (0–7)	2.2 (2.3)	1.9 (2.2)	2.2 (2.6)	2.6 (2.7)	0.074	0.029
Psycho-affective parameters						
Happiness (1–7)	5.5 (0.9)	5.7 (0.9)	5.4 (0.8)	5.3 (0.9)	0.077	0.029
Anxiety (0–9)	3.7 (2.3)	2.9 (2.4)	4.2 (2.7)	3.7 (2.7)	0.274	0.011
Depression (0–9)	2.0 (1.9)	1.5 (1.8)	2.1 (2.1)	2.0 (1.7)	**0.021**	0.049
Quality of life (0–100)	76 (13)	83 (12)	70 (16)	72 (17)	**0.002**	0.084
Sleep quality (0–10)	6.3 (2.2)	6.6 (2.3)	6.1 (1.8)	6.5 (2.0)	0.946	< 0.001
Hypnotic/anxiolytic medication (0–7)	0.8 (2.0)	0.3 (1.4)	0.9 (2.2)	1.1 (2.4)	**0.011**	0.059
Muscle performance						
5-repetition sit to stand test (s)	7.1 (1.6)	6.7 (1.3)	7.5 (2.0)	7.5 (2.2)	**0.026**	0.047
Kneeling push-up test (repetitions)	3.5 (5.1)	5.9 (6.6)	4.3 (5.5)	4.6 (6.4)	**0.031**	0.047
Shirado-Ito trunk flexor test (s)	50 (35)	58 (28)	52 (45)	50 (37)	**0.030**	0.046

Bold values are statistically significant (*p* < 0.05)

Data are mean (SD)

*s* seconds; no between-group differences in Pre (*p* > 0.05)

There were also significant group-by-time interactions in favour of the experimental group on low back pain worst intensity (*p* = 0.036), frequency (*p* = 0.003) and interference (*p* < 0.001), as well as on hand/wrist pain average intensity (*p* = 0.010), worst intensity (*p* = 0.009) and frequency (*p* = 0.023), and neck pain frequency (*p* = 0.039).

Regarding psycho-affective parameters, the group-by-time interaction was significant for depression (*p* = 0.021), quality of life (*p* = 0.002), and hypnotic/anxiolytic medication consumption (*p* = 0.011) favouring the experimental group in the three parameters.

Concerning muscle performance, there were significant group-by-time interactions favouring the experimental group in all the performed tests: 5-repetition sit to stand (*p* = 0.026), kneeling push-up (*p* = 0.031) and Shirado-Ito trunk flexor (*p* = 0.030).

There were not significant group-by-time effects on the remaining variables (*p* > 0.05).

### Intervention Effects: Post Hoc Subgroup Analysis

Among participants with low back pain at baseline, the group-by-time ANCOVA showed a significant effect of the intervention favouring the experimental group on average intensity (*p* = 0.036) (Table 4). There were also significant group-by-time interactions favouring the experimental group in low back pain frequency (*p* = 0.005) and interference (*p* = 0.001).

**Table 4:** Effects of the intervention on low back pain outcomes: post hoc subgroup analysis of participants with (≥ 1 in average NRS) and without (< 1 in average NRS) low back pain at baseline

Outcome	Experimental		Control		ANCOVA (*p*)	η_p_^2^
	Pre	Post	Pre	Post		
With low back pain^a^						
Average intensity (0–10)	4.3 (1.8)	2.9 (2.2)	3.9 (2.0)	3.5 (2.4)	**0.036**	0.046
Worst intensity (0–10)	5.2 (2.2)	3.7 (2.7)	4.7 (2.5)	4.3 (2.8)	0.121	0.025
Frequency (0–7)	3.8 (2.0)	2.3 (1.9)	3.4 (2.0)	3.3 (2.1)	**0.005**	0.080
Interference (0–7)	1.6 (2.2)	1.0 (1.7)	1.5 (2.0)	1.9 (1.8)	**0.001**	0.116
Without low back pain^b^						
Average intensity (0–10)	0.0 (0.0)	0.6 (1.0)	0.0 (0.0)	1.6 (2.5)	0.331	0.036
Worst intensity (0–10)	0.0 (0.0)	0.9 (1.4)	0.0 (0.0)	2.1 (3.0)	0.340	0.035
Frequency (0–7)	0.0 (0.0)	0.4 (0.6)	0.0 (0.0)	1.2 (2.6)	0.548	0.014
Interference (0–7)	0.0 (0.0)	0.3 (0.6)	0.0 (0.0)	1.0 (2.5)	0.646	0.008

Bold values are statistically significant (*p* < 0.05)

Data are mean (SD)

No between-group differences in Pre (*p* > 0.05)

^a^Experimental *n* = 49, Control *n* = 48

^b^Experimental *n* = 14, Control *n* = 14

There were not significant group-by-time effects of the intervention among participants without low back pain at baseline (*p* > 0.05).

## Discussion

The main result of this study is that the designed videoconference exercise intervention was effective for reducing the low back pain of eldercare workers. Improvements in hand/wrist pain and upper limb muscle performance were also observed. In the PP analysis, additional benefits were seen on neck pain frequency, depression, quality of life, hypnotic/anxiolytic medication, and the muscle performance of lower limbs and trunk. These findings provide evidence on an alternative and effective modality for delivering exercise to tackle musculoskeletal disorders. The few minor adverse events, together with the acceptable adherence and low dropout rate confirm the feasibility of the intervention proposed.

### Effects on Low Back Pain

Improvements in low back pain were consistent in both the ITT and PP analyses. Moreover, the post-hoc subgroup analysis confirmed that the intervention was also effective for the treatment of participants who already had low back pain at baseline. The low number of workers without low back pain at baseline did not allow us to draw firm conclusions regarding the preventive capacity of the intervention for the development of low back pain. While the potential for low back pain reduction of traditional in-person exercise has been well established [11], this study confirms this beneficial effect could also be achieved by videoconference-supervised group exercise. We hypothesised that this positive result might be due to the achieved volume and intensity of the intervention and the effective supervision of participants through a remote modality. Besides, it is important to note that in eldercare workers, RCTs assessing the effects of exercise on low back pain have been scarce, all limited to the face-to-face modality, and they have found conflicting results [33–36]. However, as opposed to our study, the majority of those RCTs lack an extensive description of the intervention, particularly regarding exercise content and criteria for progression or intensity adjustment, which makes it difficult to draw conclusions regarding the reasons explaining the discordant results. Reducing low back pain in eldercare workers could have a great impact, as low back pain has shown to be a significant risk factor for increased disability [37], lowered quality of life [37] and greater risk of long-term sickness absence [38] in this population.

### Effects on Neck, Shoulder and Hand/Wrist Pain

Regarding the remaining pain locations, it should be noted that the ITT analysis demonstrated improvements in all hand/wrist pain outcomes. To our knowledge, this is the first study analysing the effects of exercise on hand/wrist pain in eldercare workers. These findings are important because hand/wrist pain is highly prevalent in eldercare workers [5]. Although the hand/wrist area was not directly targeted in our exercises, it is possible that the stabilising isometric contractions of the wrist required during upper limb exercises, which have been shown to reduce wrist pain [39], were the reason for satisfactory outcomes. Concerning neck pain, improvements were only observed for pain frequency in the PP analysis, and no significant effects were found on shoulder pain. To our knowledge, the only RCT assessing the effects of an exercise intervention on neck-shoulder pain of eldercare workers was the one by Horneij et al., and they found no between-group differences [33]. Although exercise can be generally considered effective for the management of neck-shoulder disorders, interventions utilizing specific resistance training seem to obtain better results in comparison with other modalities such as general resistance training or general physical exercise [40]. Even though further research is needed [40], it is possible that our exercise program did not include enough specific neck-shoulder exercises.

### Effects on Psycho-Affective Parameters

With respect to psycho-affective parameters, although the ITT analysis did not show any significant effect of the intervention, the PP analysis demonstrated improvements in depression and quality of life and a reduction in hypnotic/anxiolytic medication use. That is, improvements in psycho-affective parameters were only observed when analysing participants with ≥ 50% adherence separately. In this regard, previous RCTs in eldercare workers in which average attendance was of only 8 [34] and 12 [41] exercise sessions (compared to 20 in our PP analysis) found no effects on depression [34] and quality of life [34, 41]. The reduction in the use of hypnotic/anxiolytic medications in the intervention group appears to be relevant because a recent prospective study with an 11-year follow-up among almost 8,000 eldercare workers found that the use of hypnotic/anxiolytic/sedative medication increased the risk of disability pension and mortality [42]. In addition, keeping a good psychological health of eldercare workers could be important not only for ensuring their wellbeing but also for guaranteeing a high-quality care for the older people. In this regard, previous research has shown that a higher caregiver burden predicts a greater hospitalization risk of the older person [43], and burnout symptoms in nurses are related to lower quality of care [44].

### Effects on Muscle Performance

Regarding muscle performance, although the ITT analysis only showed a significant improvement in upper limb performance, trunk and lower limb performance also improved in the PP analysis. From a biomechanical point of view, current literature supports the idea that improvements in the structure and function of the musculoskeletal system, specially muscle strength, could contribute to the pain reduction induced by exercise [45]. While improvements in muscle performance may seem obvious and expected in exercise trials, their potential impact should not be neglected. In the context of this study, a higher physical capacity could permit eldercare workers confront their daily tasks in a less strenuous and safer manner, therefore reducing the risk of suffering an injury or developing/increasing pain. As a consequence, a better balance between intrinsic personal resources and extrinsic job demands could be achieved, what could lead to a better health state of the workers [46].

### Strengths and Limitations

The main strength of the present study lies in its RCT design with outcome assessor and data analyst blinding, as well as a proper sample size calculation. Study procedures and intervention characteristics are thoroughly described, allowing easy replicability. Moreover, the unrestrictive selection criteria, together with the simple exercises and few cheap materials used, confer the study a pragmatic nature. This allows the results be generalisable to what could happen in a real-world setting and facilitates scalability, what has been asserted as a priority in physical activity research [47]. However, it should be admitted that this could not apply to world regions in which access to the Internet and videoconference technologies is not yet widespread. Regarding participant retention throughout the study, the low dropout rate suggests high acceptability and feasibility of the intervention. In this sense, and based on the technical adverse events found, ensuring a high-quality connection and a good familiarisation with technology seems key for a satisfactory delivery of exercise via videoconference. Regarding outcomes, pain was studied from a broad perspective, including diverse pain characteristics and locations, as well as other pain-related variables such as medication, psycho-affective parameters and muscle performance. Despite their key contribution to pain from a biopsychosocial point of view, these variables have been understudied in low back pain research, and their inclusion in future exercise trials has been urged [48]. Finally, we only included eldercare workers. This could be important because most exercise trials in this population have merged other professionals such as nurses, physiotherapists or midwives in their samples [33–36, 41], what limits the applicability of the findings to the eldercare workers.

On the other hand, some limitations should be acknowledged. For example, despite the unrestrictive selection criteria, the participation rate was of only 30% (130 out of 436 participants assessed for eligibility). This could be relevant because the characteristics of the participants could differ from those who rejected participation and therefore the effects of the intervention might not be the same in the latter. One possible reason to explain our low participation rate could be that the exercise program was carried out outside working hours, what was found to be one of the main barriers for participation in a health promotion program in healthcare workers [49]. Apart from rising participation rates, the additional benefits observed in the PP analysis make evident the necessity of finding strategies to improve adherence. With respect to the PP analysis, it should be acknowledged that the randomisation effect is lost due to excluding some participants based on their level of adherence to the intervention. Therefore, although one may tend to attribute the additional improvements found in the PP analysis to the higher adherence, it cannot be ruled out that the participants with ≥ 50% or < 50% adherence had different characteristics at baseline (e.g., motivation or expectation to improve) and that these could have interfered in the findings observed. Also, the inherent impossibility of exercise trials to blind participants could have biased the results obtained. Regarding statistical power, the secondary outcomes of the present study could be not powered enough, and new studies might be necessary to draw more reliable conclusions. Besides, the single-item sleep quality scale used in the present study has not been cross-culturally validated in Spanish yet. Although it is a numerical scale containing minimum text, it should be acknowledged that we translated it from the original English version. Finally, although including only eldercare workers can be considered a strength, it should also be recognised that due to the specific characteristics of the sample, the findings of this study could not be directly applicable to other populations. Similarly, the great majority of female participants in the present study, while reflective of the reality of the eldercare sector, could also limit the applicability of the findings to male eldercare workers.

## Conclusions

The group exercise intervention carried out by real-time videoconference was effective for reducing the low back pain of eldercare workers. Improvements in hand/wrist pain and upper limb muscle performance were also observed. The results from the PP analysis suggest that a higher adherence to the intervention could lead to additional benefits in psycho-affective parameters, medication consumption and muscle performance. To our knowledge, this is the first group exercise intervention conducted by videoconference in the workplace, which provides an evidence-based alternative modality of exercise delivery to tackle musculoskeletal disorders.

## Supplementary Information

Below is the link to the electronic supplementary material.

Fig. SI1 Setting up of the real-time videoconference exercise sessions. a: screenshot of a videoconference session, b: setting of the instructor, c: setting of participants in a nursing home. Adapted from: Espin et al., 2023 (doi: 10.1186/s12891-023-06584-7)
Supplementary file1 (TIF 1734 KB)

Fig. SI2 Exercises performed throughout the program. kg: kilograms, s: seconds. Adapted from: Espin et al., 2023 (doi: 10.1186/s12891-023-06584-7)
Supplementary file2 (TIF 1691 KB)

Fig. SI3 Progression of the work:rest time ratio devoted to each exercise throughout the program. s: seconds, wk: week. Adapted from: Espin et al., 2023 (doi: 10.1186/s12891-023-06584-7)
Supplementary file3 (TIF 188 KB)

Table SI1 Summary of the effect sizes of the outcomes showing statistically significant group-by-time interactions
Supplementary file4 (DOCX 18 KB)

## Data Availability

The data that support the findings of this study are available from the corresponding author upon request.
