# Supplementary figures and images for: Videoconference-Supervised Group Exercise Reduces Low Back Pain in Eldercare Workers: Results from the ReViEEW Randomised Controlled Trial
Source: J Occup Rehabil. 2024 Apr 17;35(1):66–77. doi: 10.1007/s10926-024-10182-2 (PMC11839872; doi:10.1007/s10926-024-10182-2)

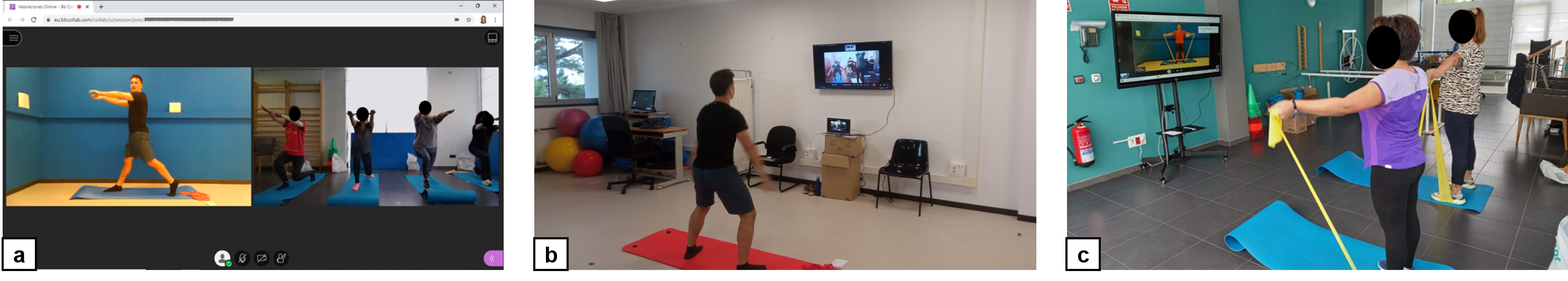

Supplement: Supplementary file 1 — Fig. SI1 Setting up of the real-time videoconference exercise sessions. a: screenshot of a videoconference session, b: setting of the instructor, c: setting of participants in a nursing home. Adapted from: Espin et al., 2023 (doi: 10.1186/s12891-023-06584-7) Supplementary file1 (TIF 1734 KB) [file 10926_2024_10182_MOESM1_ESM.tif]

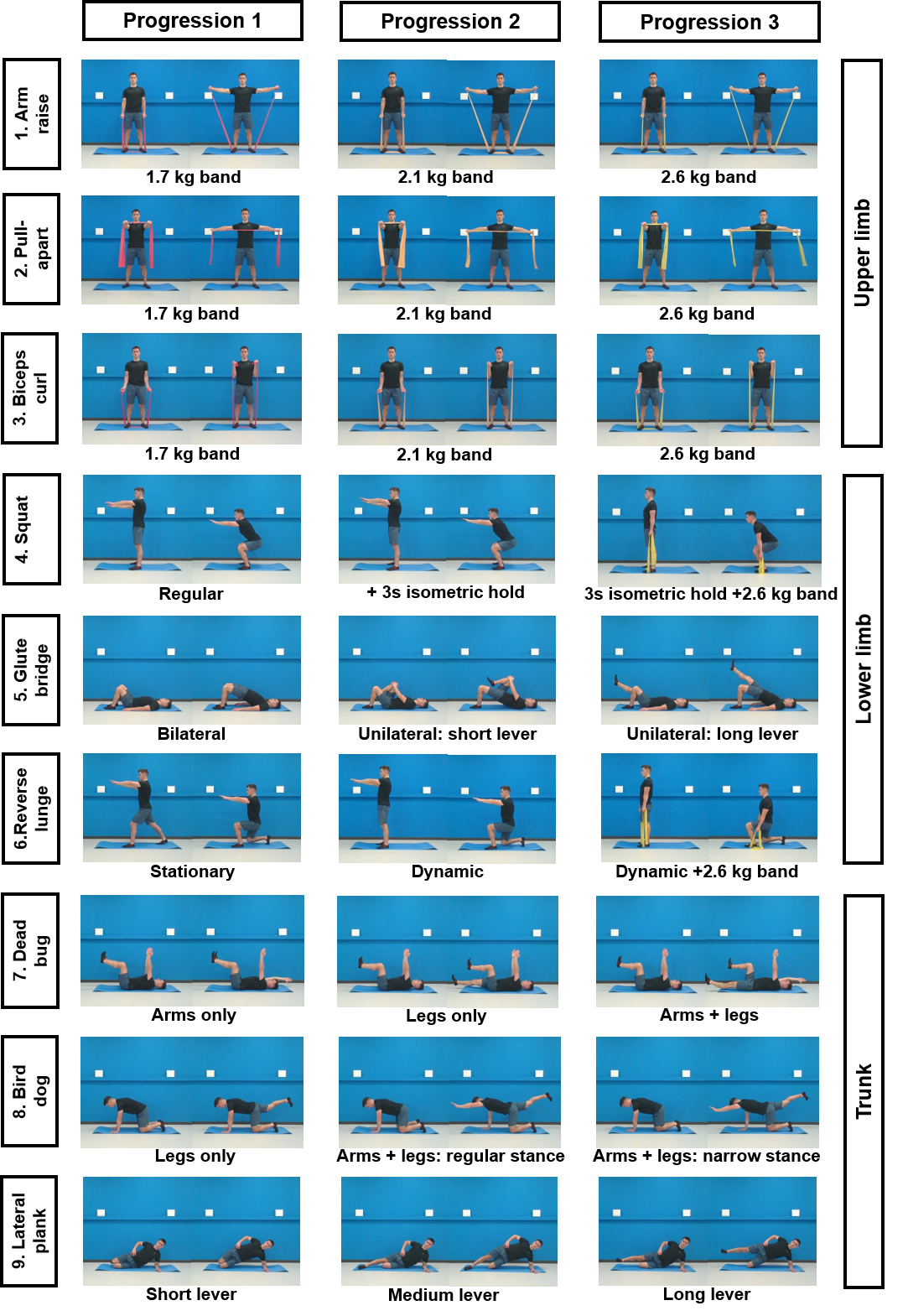

Supplement: Supplementary file 2 — Fig. SI2 Exercises performed throughout the program. kg: kilograms, s: seconds. Adapted from: Espin et al., 2023 (doi: 10.1186/s12891-023-06584-7) Supplementary file2 (TIF 1691 KB) [file 10926_2024_10182_MOESM2_ESM.tif]

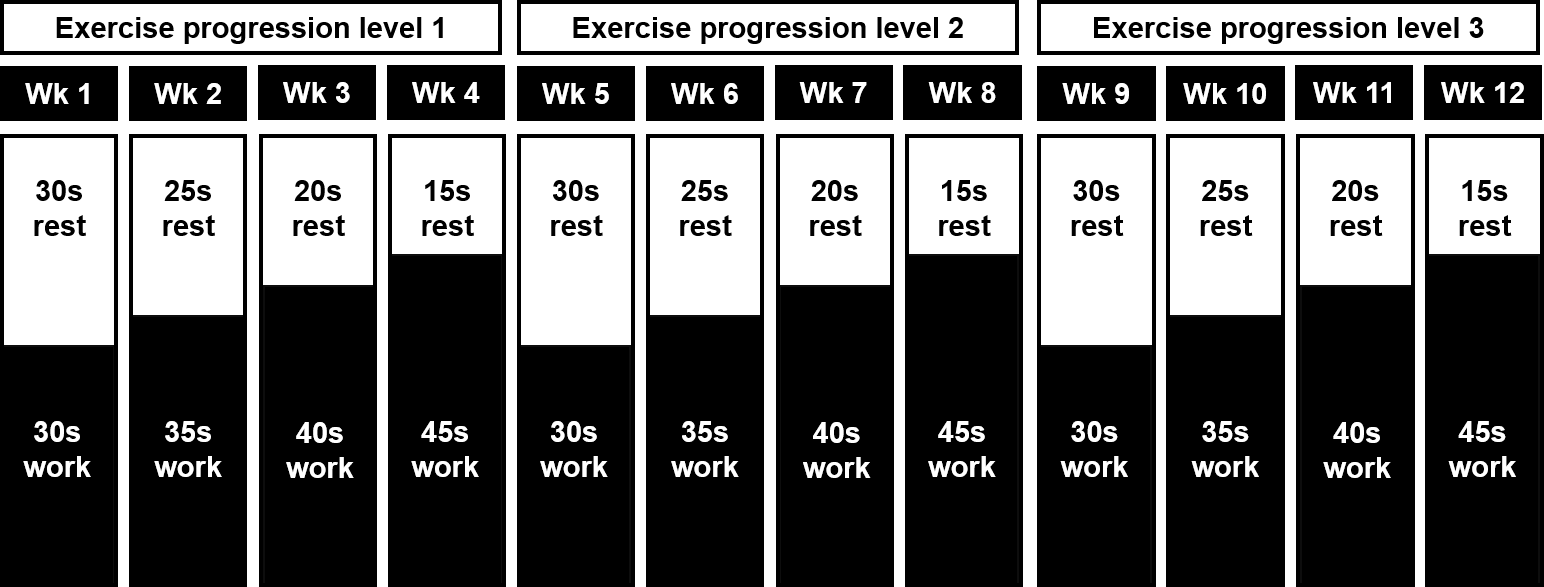

Supplement: Supplementary file 3 — Fig. SI3 Progression of the work:rest time ratio devoted to each exercise throughout the program. s: seconds, wk: week. Adapted from: Espin et al., 2023 (doi: 10.1186/s12891-023-06584-7) Supplementary file3 (TIF 188 KB) [file 10926_2024_10182_MOESM3_ESM.tif]
