# Supplementary material for: Videoconference-Supervised Group Exercise Reduces Low Back Pain in Eldercare Workers: Results from the ReViEEW Randomised Controlled Trial
Source: J Occup Rehabil. 2024 Apr 17;35(1):66–77. doi: 10.1007/s10926-024-10182-2 (PMC11839872; doi:10.1007/s10926-024-10182-2)
Supplement: Supplementary file 4 — Table SI1 Summary of the effect sizes of the outcomes showing statistically significant group-by-time interactions Supplementary file4 (DOCX 18 KB) [file 10926_2024_10182_MOESM4_ESM.docx]

| **Outcome** | **ITT analysis** | **PP analysis** | **Post-hoc analysis:**  **with LBP** | **Post-hoc analysis: without LBP** |
| --- | --- | --- | --- | --- |
| Low back pain |  |  |  |  |
| Average intensity | Medium | Medium | Medium | n.s. |
| Worst intensity | n.s. | Medium | n.s. | n.s. |
| Frequency | Medium | Medium | Medium | n.s. |
| Interference | Medium | Large | Large | n.s. |
| Neck pain |  |  |  |  |
| Average intensity | n.s. | n.s. | n.a. | n.a. |
| Worst intensity | n.s. | n.s. | n.a. | n.a. |
| Frequency | n.s. | Medium | n.a. | n.a. |
| Interference | n.s. | n.s. | n.a. | n.a. |
| Shoulder pain |  |  |  |  |
| Average intensity | n.s. | n.s. | n.a. | n.a. |
| Worst intensity | n.s. | n.s. | n.a. | n.a. |
| Frequency | n.s. | n.s. | n.a. | n.a. |
| Interference | n.s. | n.s. | n.a. | n.a. |
| Hand/wrist pain |  |  |  |  |
| Average intensity | Medium | Medium | n.a. | n.a. |
| Worst intensity | Medium | Medium | n.a. | n.a. |
| Frequency | Medium | Medium | n.a. | n.a. |
| Interference | Small | n.s. | n.a. | n.a. |
| Pain medication | n.s. | n.s. | n.a. | n.a. |
| Psycho-affective parameters |  |  |  |  |
| Happiness | n.s. | n.s. | n.a. | n.a. |
| Anxiety | n.s. | n.s. | n.a. | n.a. |
| Depression | n.s. | Medium | n.a. | n.a. |
| Quality of life | n.s. | Medium | n.a. | n.a. |
| Sleep quality | n.s. | n.s. | n.a. | n.a. |
| Hypnotic/anxiolytic medication | n.s. | Medium | n.a. | n.a. |
| Muscle performance |  |  |  |  |
| 5-repetition sit to stand test | n.s. | Medium | n.a. | n.a. |
| Kneeling push-up test | Medium | Medium | n.a. | n.a. |
| Shirado-Ito trunk flexor test | n.s. | Medium | n.a. | n.a. |

Effect sizes based on η_p_^2^ (0.01=small; 0.06=medium; 0.14=large) ^1^

ITT: intention-to-treat

PP: per-protocol

LBP: low back pain

n.s.: non-statistically significant group-by-time interaction (p<0.05 in ANCOVA)

n.a.: not analysed group-by-time interaction

^1^ Richardson JTE. Eta squared and partial eta squared as measures of effect size in educational research. Educ Res Rev. 2011;6(2):135–47.
